# Design of infrared optical absorber using silver nanorings array made by a top-down process

**DOI:** 10.1038/s41598-023-34579-w

**Published:** 2023-05-12

**Authors:** I. Bouanane, F. Bedu, I. Ozerov, B. Sciacca, L. Santinacci, D. Duché, G. Berginc, L. Escoubas, O. Margeat, J. Le Rouzo

**Affiliations:** 1 Aix Marseille University, Université de Toulon, CNRS, IM2NP, Marseille, France; 2 Thales LAS France SAS, Élancourt, France; 3 Aix Marseille University, CNRS, CINAM, AMUTECH, Marseille, France

**Keywords:** Metamaterials, Sub-wavelength optics

## Abstract

This paper presents the numerical simulation and fabrication of a metasurface composed of silver nanorings with a split-ring gap. These nanostructures can exhibit optically-induced magnetic responses with unique possibilities to control absorption at optical frequencies. The absorption coefficient of the silver nanoring was optimized by performing a parametric study with Finite Difference Time Domain (FDTD) simulations. The absorption and scattering cross sections of the nanostructures are numerically calculated to assess the impact of the inner and outer radii, the thickness and the split-ring gap of one nanoring, as well as the periodicity factor for a group of four nanorings. This showed full control on resonance peaks and absorption enhancement in the near infrared spectral range. The experimental fabrication of this metasurface made of an array of silver nanorings is achieved by e-beam lithography and metallization. Optical characterizations are then carried out and compared to the numerical simulations. In contrast to usual microwave split-ring resonator metasurfaces reported in literature, the present study shows both the realization by a top-down process and modelling performed in the infrared frequency range.

## Introduction

The design of absorbing metasurfaces in the visible and infrared ranges is key in various domains such as solar thermal and photovoltaics, optoelectronics (photodetectors, sensors, etc.) or even for functional materials requiring selective absorbers^1–7^. The opportunity to employ sub-wavelength nanostructures to tune light-matter interaction has aroused tremendous interest during the last decade, thanks to the plethora of unique optical properties that can be achieved with metamaterials or metasurfaces, that are not seen in nature by definition^8–14^. Among those possibilities, metallic split-ring resonators are well-known metamaterials allowing unique possibilities for controlling the response to the electrical and magnetic components of light in visible^15–17^ and infrared bands [near infrared (NIR) and short-wave infrared (SWIR)]^18–20^. In addition to resonances with electric character, metallic split-ring resonators support optically-induced magnetic resonances as a result of their circular shape^21–26^. Electric and magnetic fields can be enhanced and consequently cause an enhancement of absorption properties at the resonance wavelength^19,27,28^. In addition, split-ring resonators allow unique possibilities for precisely controlling the light response depending on their geometric parameters. The nanoring design provides a great platform to easily tune the plasmonic resonance properties thanks to a precise set of parameters: the inner and outer radii, the split-ring gap and the structure thickness^29,30^. Several technological approaches have been studied for the realization of such split-ring resonators^17,31–33^. For instance, we recently proposed an innovative bottom-up approach^34^ for the fabrication of nanophotonic surfaces from colloidal nanocube building blocks, showing the potential to be a game-changer in metasurface low cost fabrication^35,36^. This previous study allowed us to define restrictions on the metasurface design that is not considered in previous reports^37,38^. These technological constraints have been considered in this study, which this time uses a top-down process. Here, the fabrication of the silver nanorings array is achieved by e-beam lithography and metallization. The objective is to optimize the metasurface design thanks to FDTD simulations and validate the model thanks to a well-known reliable fabrication method. Consequently, both FDTD simulation and top-down fabrication are done by considering design constraints. We propose a set of realistic parameters of nanorings arrays to achieve an absorption enhancement in the near-infrared range (λ = 1000–2000 nm) that can be attractive for a large number of optoelectronic applications^39–41^. Silver is chosen as the constituent material due to its excellent optical and electronic properties^42^.

First, we provide with various optimum designs that maximize the absorptance of the split-ring arrays at different infrared frequencies, and we compare the response of individual resonators to that of arrays; next, we fabricate the metasurfaces by standard top-down process on a transparent substrate. Finally, we characterize the optical properties of the metasurfaces and compare them to numerical simulations to validate the designs. This study shows the simulation and the top down fabrication of an optimized metasurface made of silver nanorings, exhibiting a great exaltation of its absorption properties at a specific wavelength of 1500 nm while taking into account the technological constraints.

## Results

### Silver nanorings design and modelling

**Figure 1:**
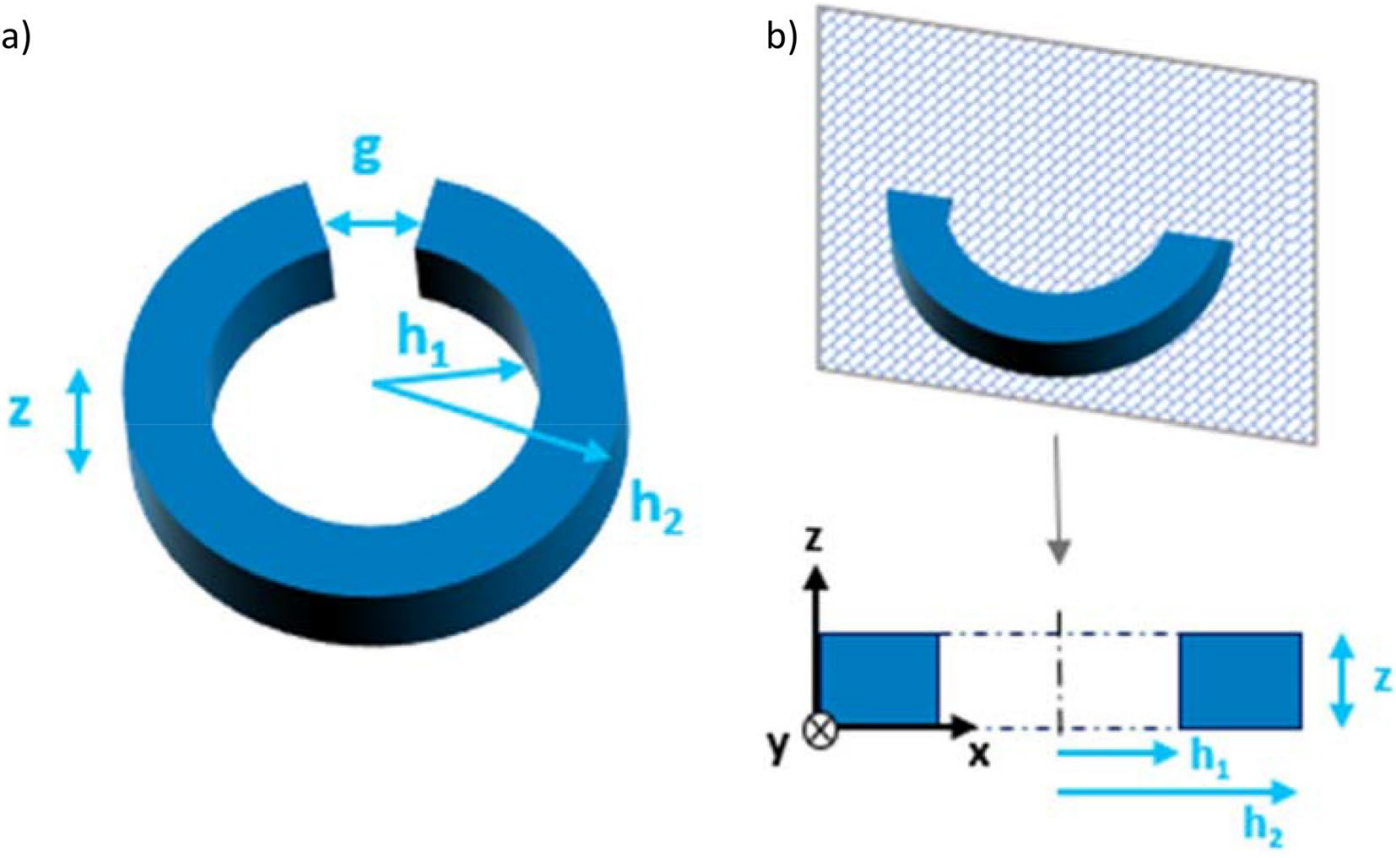
(**a**) Schematic of the silver nanoring structure. The inner radius of the ring is labeled *h*_1_, the outer radius *h*_2_, the thickness *z* and the split-ring gap *g*. (**b**) Cross-section view of the nanoring.

The impact of the geometrical properties on the optical response is investigated by performing a parametric study via Finite Difference Time Domain (FDTD) simulations (see Figures S1, S2 and “ Methods” section for further details)^43–48^. The absorption and scattering cross-section for an individual split-ring are calculated as a function of inner (*h*_1_) and outer (*h*_2_) radii, thickness (*z*) and split-ring gap (*g*), described in Fig. 1, by varying one parameter at a time. The fabrication constraints are implemented by setting a square cross-section (z = h_2_ − h_1_). The extinction efficiency is computed from the scattering and absorption cross sections. Maximization of absorption cross-section at 1500 nm is used as the figure of merit to select the optimal geometry of the split-ring. This is then used as a fixed parameter to optimize the absorption cross-section of 4 coupled split-ring, as a function of the distance.

**Figure 2:**
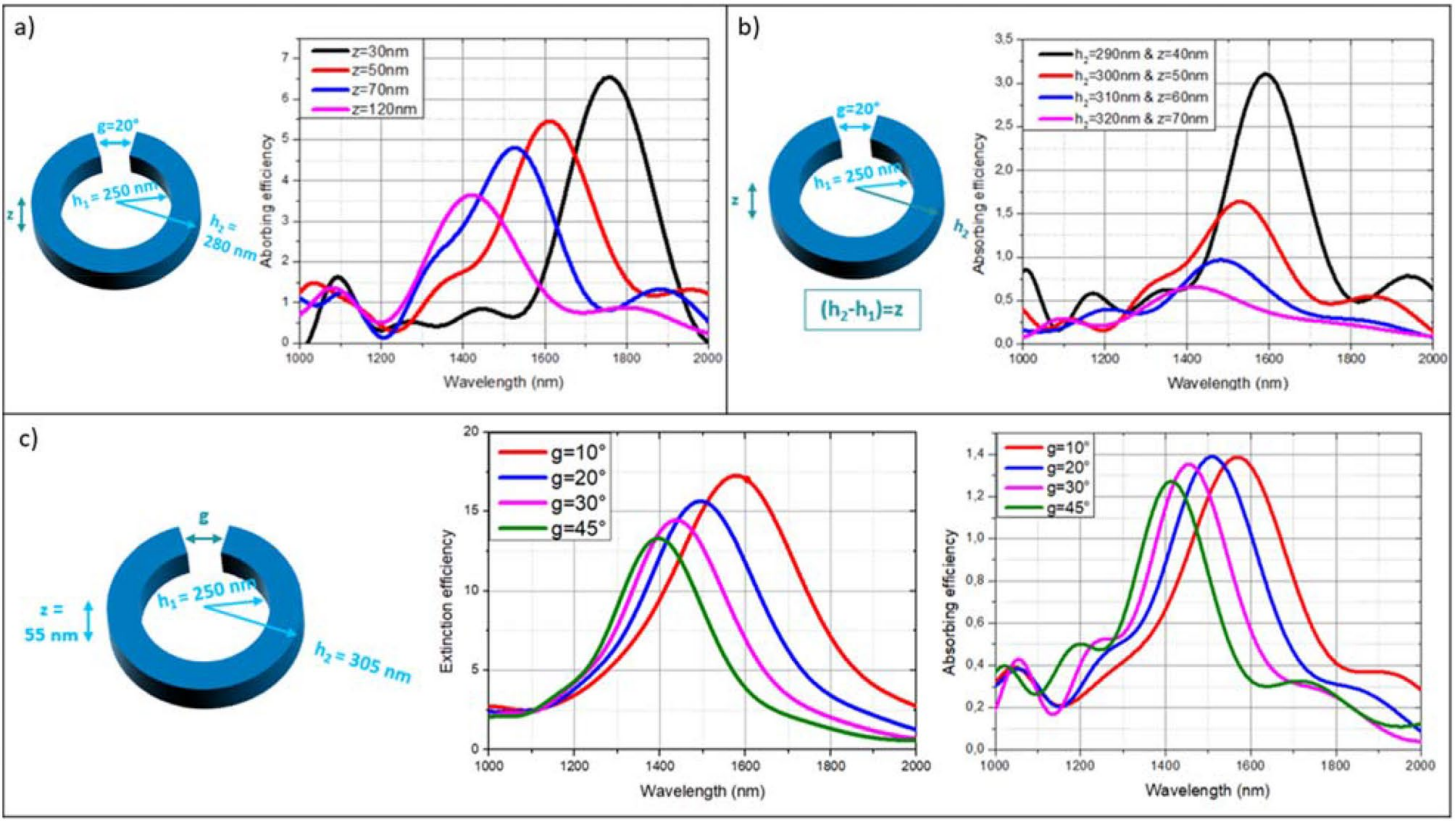
Wavelength-dependent absorbing efficiency of the structure presented on the left of each diagram. (**a**) The thickness only being varied. (**b**) The thickness equaling the width of the nanoring being varied. (**c**) The split-ring gap being varied and the wavelength-dependent extinction and absorbing efficiencies of the structure presented.

Figure 2 illustrates the impact of geometrical parameters on the cross-section. Several peaks are observed corresponding to high-order plasmonic resonance modes as reported in literature^19^. Increasing the inner radius *h*_1_ leads to a red-shift of the extinction efficiency Q_ext_ and the increase of the scattering and absorption efficiencies. Conversely, the increase in thickness *z* and split-ring gap *g* results in a smaller Q_ext_ and a blue-shift^34^. Figure 2 focuses on the wavelength-dependent absorbing efficiency Q_abs_ of the structure. Q_abs_ is the normalized absorption cross section (i.e. σ_abs_/A) where A is the projected cross-sectional area of the nanoring in the x–y plane. A preliminary study allowed to define the fixed parameters to achieve wavelength resonances between 1000 and 2000 nm. The variation of z only has nevertheless been studied, with an inner radius h_1_ of 250 nm, an outer radius h_2_ of 305 nm and a gap g of 20°. It appears in Fig. 2a that the lower the thickness of the ring z varies, here from 120 to 30 nm, the higher is the absorption efficiency. When the z parameter is being varied from 40 to 70 nm, considering this time the constraint of fabrication, meaning varying z as well as (h_2_ − h_1_), h_1_ held constant at 250 nm, as illustrated in Fig. 2b. It shows that the more it increases, the more the absorbing efficiency decreases in addition to a blue-shift trend for the resonance wavelength due to the z parameter, as well as a widen of the Q_abs_ peak due to the *h*_2_ variation. The z parameter can therefore be used as an alternative parameter in tuning the values of the optical properties and the resonance wavelengths. Moreover, these first two figures show a better absorption in the case of z independent of (h_2_ − h_1_) value. Therefore, the fabrication constraints do not allow to choose the best performing absorption value for the wavelength of interest of 1500 nm but the design will be optimized according to the geometric parameters suitable for the fabrication.

Another important parameter is g. Figure 2c shows the extinction efficiency on the left and the absorption efficiency on the right of one single nanoring by varying the angle g from 45° to 10°. It is observed that the more g decreases, the more the extinction efficiency increases, consequently the scattering efficiency, but, above all, the more the absorbing efficiency increases to some extent for the gap angles calculated here. Indeed, for the structure calculated, *h*_1_ = 250 nm, *h*_2_ = 305 nm and *z* = 55 nm, Q_abs_ increases up to a g value of 20° before remaining the same while g decreases to 10°. A gap angle of 10° equals approximately to a distance of 50 nm which corresponds to an experimental limit fixed in order to assure a good realization as shown in silver nanorings fabrication of “Results” section. The evolution of both the resonant frequency and the absorbing efficiency, as function of all these geometrical parameters are summarized with polynomial regressing fitting in Figure S3 of the Supplementary Note.

Taking into consideration all the experimental constraints, the results of this parametric study allowed to define a set of parameters of an individual silver nanoring, suitable for fabrication and showing enhancement of the absorption properties at a wavelength of 1500 nm. The pattern that will therefore make up the experimental metasurface and whose periodicity will be studied below, has the following geometry: *h*_1_ = 250 nm, *h*_2_ = 305 nm and *z* = 55 nm and *g* = 20°.

**Figure 3:**
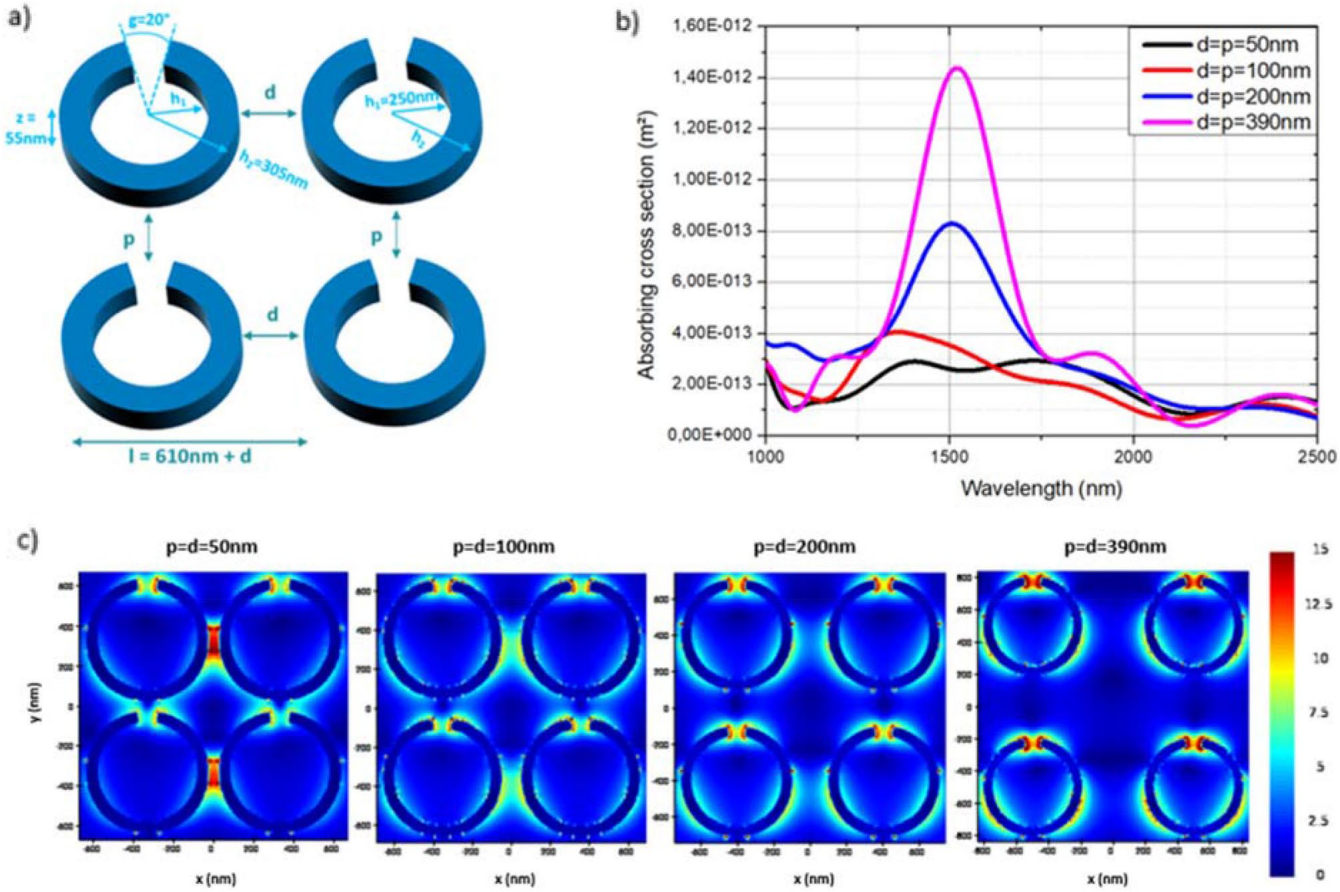
(**a**) Structure of the nanorings studied with d and p the free space distances in the x and y axes. (**b**) Wavelength-dependent absorption cross sections of four silver nanorings with the free space distances, d being varied in the x axis and p being varied in the y axis, both from 50 to 390 nm and (**c**) Mapping of the electric field intensity at the wavelength of resonance of each group of four nanorings studied.

Once the parameters of the individual ring allowing absorption at the wavelength of interest of 1500 nm have been defined, the impact of the free-space distance between the nanorings is studied. The scattering and absorption cross sections have been simulated with a x-polarized electric field, for the geometric parameters mentioned above. As illustrated in Fig. 3, the optical properties of four nanorings thereby are studied in order to evaluate the impact of the free-space distance in the x-axis *d* but also in the y-axis *p*. The structure, presented Fig. 3a, is composed of four nanorings exhibiting the geometry defined above. Both *d* and *p* are varied, by being equal with each other, from 50 to 390 nm. The more d = p increases, the more the absorbing cross section increases (Fig. 3b). Indeed, Fig. 3b shows a great exaltation of the absorption cross section (more than three times greater) at the wavelength of 1500 nm for four silver nanorings spaced by 390 nm (in pink) in the x and y axes, compared to four nanorings spaced by 50 nm (in black). Although the coupling between the nanorings (Fig. 3c) seems more important when *d* is low, the mapping shows a stronger field concentration at the split-ring gap *g* despite the distance when d = 390 nm. Interestingly, it appears that the rings resonate at this periodicity allowing an enhancement of the absorption response.

**Figure 4:**
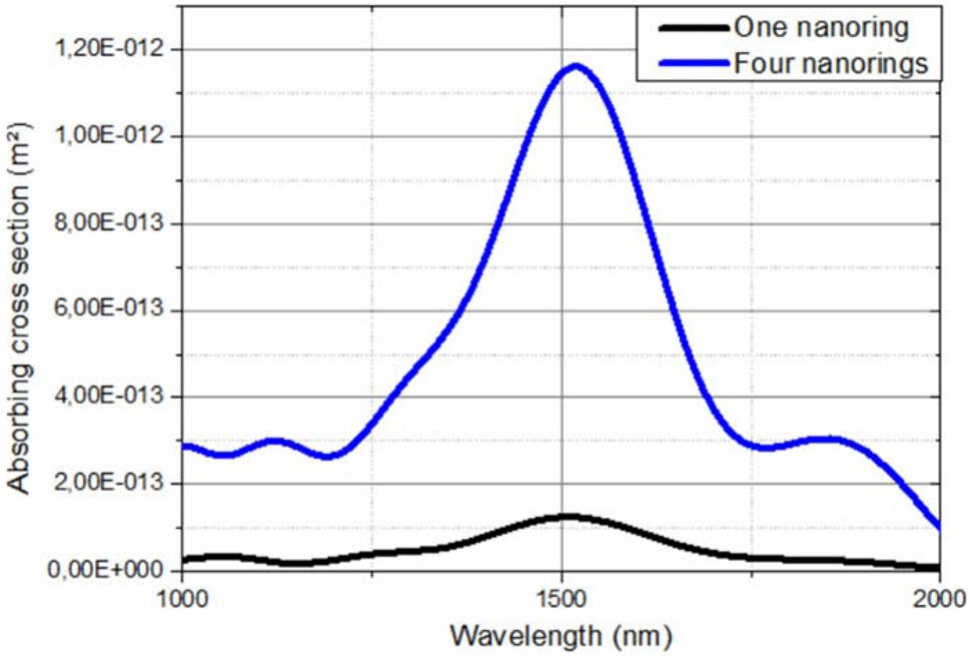
Wavelength-dependent absorption cross section of a single nanoring in black and four nanorings with a free space distance of 390 nm in the x and y axes in blue. The parameters of the single nanoring used are shown in Fig. 1.

Consequently, thanks to the parametric study and the impact study of the free-space distance for four nanorings, the choice of the pattern to be fabricated could be made. The metasurface that will be fabricated is composed of an array of silver nanorings, each having an inner radius *h*_1_ of 250 nm, an outer radius *h*_2_ of 305 nm, a thickness *z* of 55 nm, a split-ring gap *g* of 20° and being spaced from each other by 390 nm. The comparison of the absorbing cross section of a single (in black) and four (in blue) nanorings in the Fig. 4, shows a significant exaltation of the absorbing efficiency of three times for four nanorings compared to one single nanoring. These results confirmed the choice of the structure as it suggests, indeed, even more exalted absorption properties with an ordered array of nanorings, and justifies the interest of realizing this metasurface pattern. Finally, we investigate the light polarization dependence of the chosen pattern. The effect of the electric field polarization is presented in the Figure S4 of the Supplementary Note. The absorption and scattering cross sections (Fig. S4a,b respectively) have been simulated with an electric field oriented parallel (in black) and perpendicular (in red) to the x-axis, *E*_//_ and *E*__|__ respectively. When the electric field is tilted from the parallel polarization to the perpendicular one, an enhancement of the extinction cross section as well as a blue-shift of the resonance wavelength are observed. Indeed, the scattering cross section increases when the absorbing cross section decreases. A great enhancement of the electric field is observed at the nanorings gap *g* for the parallel polarization unlike the perpendicular polarization as shown on the mapping of the electric field intensity shows (Fig. S4c,d).

### Silver nanorings fabrication

**Figure 5:**
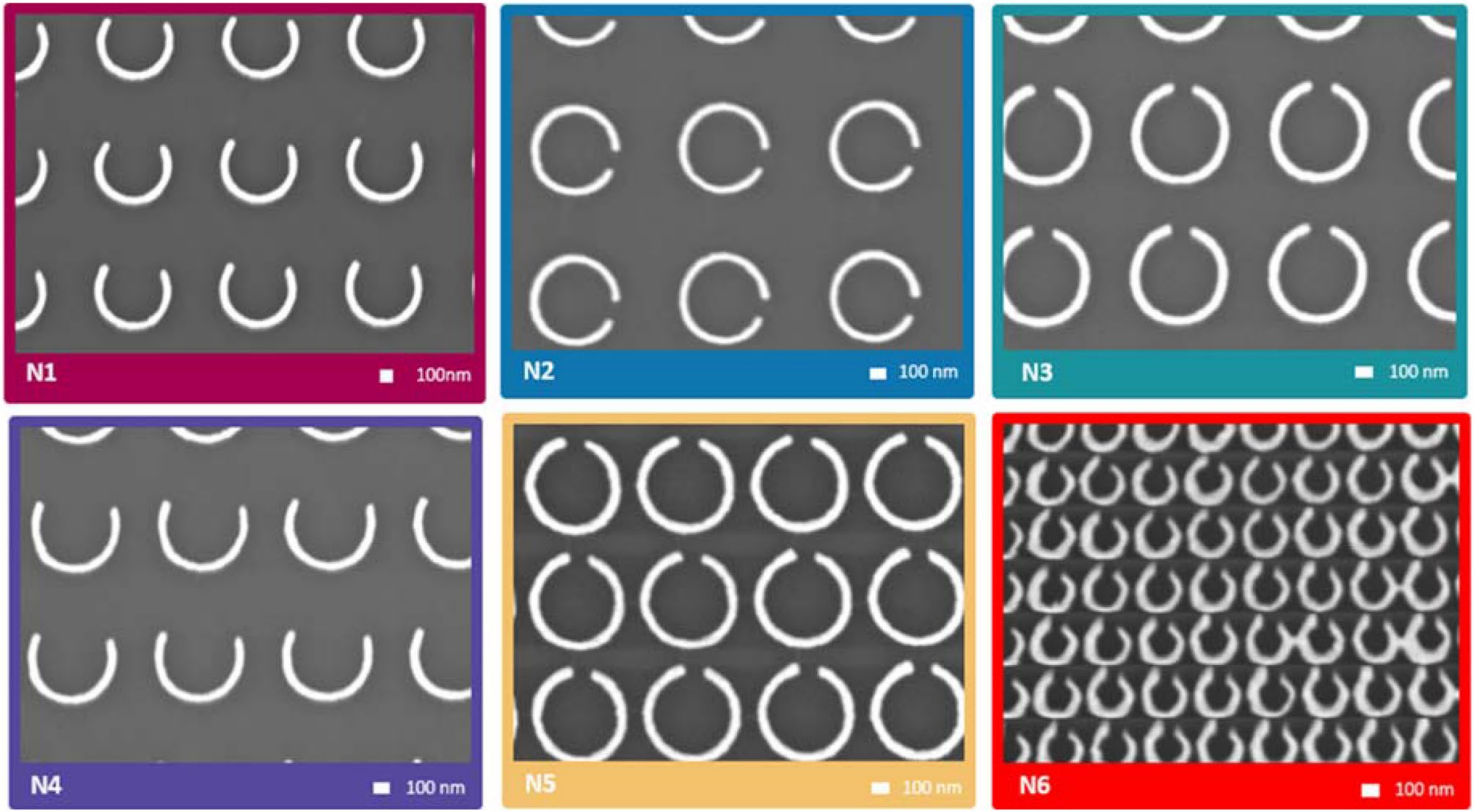
SEM top views of the silver nanorings arrays exhibiting various parameters fabricated designs in the different areas.

The fabrication is here made by a classic top-down process. Indeed, since the nanotechnology boom of the past decades, different techniques of nanofabrication have emerged^49–51^. Among these numerous processes developed, the nanolithography and especially the electron-beam lithography is a top-down method that has been most widely implemented for producing sub-micron-sized features^49,52–57^. It is now a well-known technique that is widely used to develop various metasurfaces. Consequently, to fabricate the patterns presented in Fig. 5, we used the e-beam lithography process followed by evaporation of the silver material. Those different patterns correspond to the size scale of the optimized structure allowing an absorption in near IR bands.

Six different silver nanorings arrays of 2 mm^2^ each of various parameters (N1–N6) are arranged on a single transparent substrate of 2 in. diameter. Their specific parameters are summarized in the Table 1. According to the modelling presented above, different configurations were tested to assess the validity of the study. Thus, the width (*h*_2_ − *h*_1_) and the split-ring gap *g* have been varied as well as the periodicity (l = 2 × *h*_2_ + *d*) of the array. The main steps of the fabrication method are summarized Figures S5 and S6 (see also Experimental section for more details).

**Table 1:** Summary of the geometric parameters of each area of silver nanorings arrays.

	N1	N2	N3	N4	N5	N6
h_1_	250 nm	250 nm	250 nm	250 nm	250 nm	100 nm
h_2_	305 nm	305 nm	305 nm	305 nm	305 nm	140 nm
h_2_–h_1_	55 nm	55 nm	55 nm	55 nm	55 nm	40 nm
g	100°	20°	20°	120°	20°	30°
d = p	390 nm	390 nm	250 nm	250 nm	100 nm	50 nm
l	1 µm	1 µm	0.860 µm	0.860 µm	0.710 µm	0.330 µm

The SEM images in Fig. 5 shows the successful results of the metasurface fabrication with six areas of silver nanorings arrays composed by several different geometric parameters. With the N5 area, which corresponds to rings spaced by 100 nm each, it can be seen that despite the small spacing, the quality of the rings is very good, the geometry parameters being respected. However, the N6 zone, which corresponds to smaller rings with a periodicity of 50 nm, shows that the limits of this manufacturing process via e-beam lithography are reached. In addition to the SEM images, the thickness of the nanorings was verified by atomic force microscopy (AFM) as shown in Figure S7. The thickness measured is about 39 nm which corresponds approximately to the thicknesses deposited on the substrate, which are 35 nm of silver and 3 nm of chromium. The fabrication procedure is therefore considered to be satisfactory.

**Figure 6:**
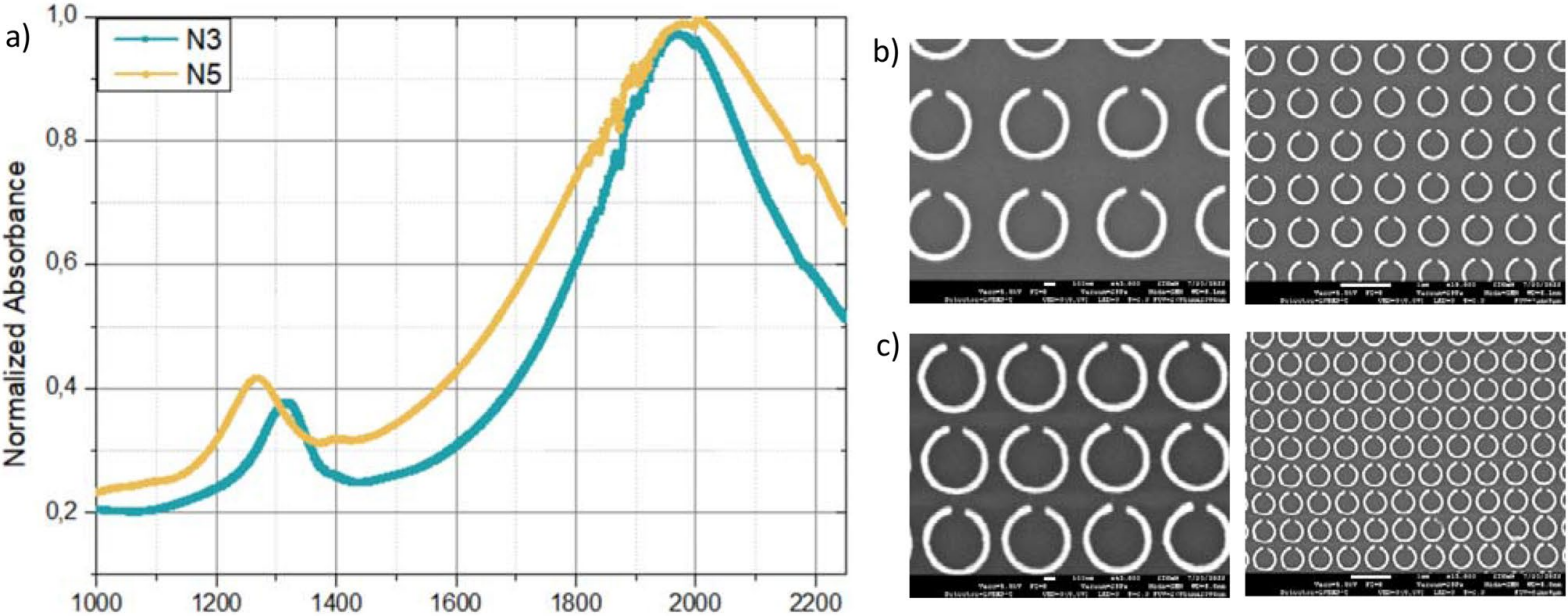
(**a**) Wavelength-dependent normalized absorption measured for N3 and N5 zones. SEM images of the arrays N3 (**b**) and N5 (**c**).

Once the nanorings arrays were obtained, the different zones were characterized optically using a spectrophotometer. Figure S8 shows an example of a large surface obtained on the N5 array of more than 26 × 20 µm^2^ without any periodicity errors, necessary for carrying out the measures. We focus our experimental work of performed measurement on two different zones, N3 and N5, as shown with the normalized absorption measured in Fig. 6. Between the two areas, only the periodicity changes as the nanorings of the zone N3 are spaced by 250 nm whereas spaced by 100 nm in the area N5. The biggest resonance peaks seem to remain the same whereas the small one shifts by less than 100 nm from 1230 nm for N5 (d = p = 100 nm) to 1315 nm for N3 (d = p = 250 nm). Importantly, a large red-shift of the resonance wavelength is noticed compared to the simulation studies performed above. This is due to the presence of the substrate that was not included in the simulations. This red-shift is indeed confirmed, as shown in Figure S9, where the optical properties of four nanorings in vacuum are compared to those of four nanorings with a thin layer of chromium and deposited on a substrate of glass.

According to literature^58^, a thin layer of alumina (Al_2_O_3_) has been deposited in order to protect the evaporated silver samples from air contamination. A layer of 3 nm thick has been deposited by atomic layer deposition to see the impact on the absorption peak, and compared to a thickness of 10 nm. The normalized absorptions were measured by spectrophotometer for the two areas N3 and N5 in Figure S10, without any layer of alumina and with a layer of 3 nm and 10 nm, respectively. It shows an absorption peak shift of about 50 nm after each deposit. For N3, we observe a 58 nm shift peak after deposition of 3 nm of Al_2_O_3_ and 112 nm in total after a 10 nm thick layer. For N5, a 32 nm shift peak is found after a deposition of 3 nm of Al_2_O_3_ and 120 nm in total after a 10 nm thick layer. In conclusion, a deposition of a thin layer of alumina allows to protect the metasurface while having a very slight impact on the optical response.

**Figure 7:**
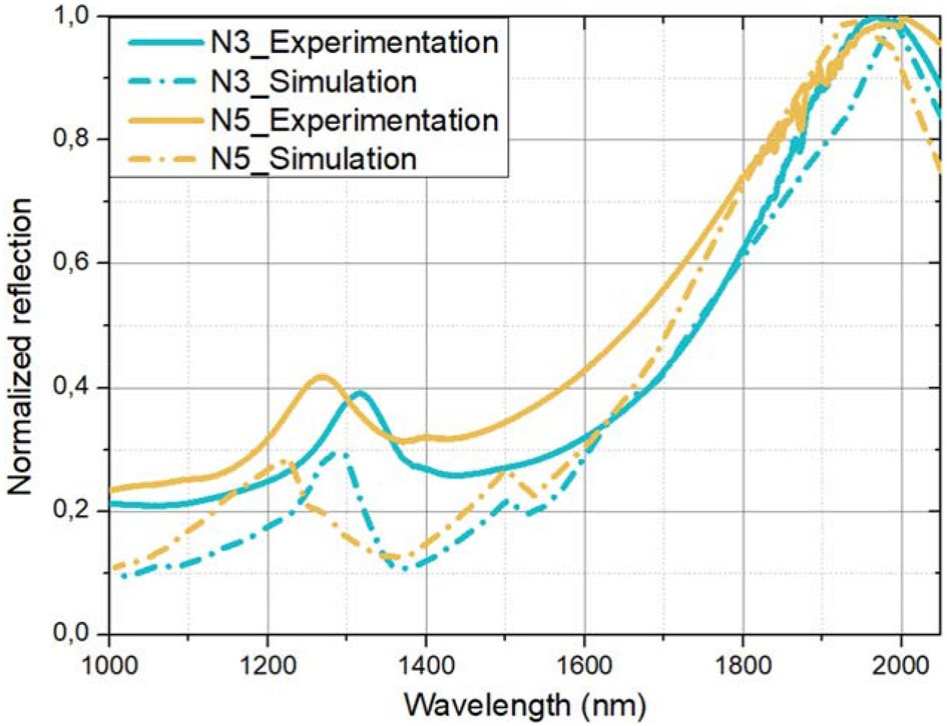
Normalized reflection spectra of the N3 and N5 zones measured by spectrophotometer on for the fabricated design (in solid line) and the corresponding numerically simulated ones (in dotted line).

To compare simulations results with experimental characterizations and therefore validate the model of simulation, the optical responses of the different zones of the metasurface have been simulated thanks to the computation configuration presented in the first section and schematized in Figure S2. Figure 7 focuses on the N3 zone in blue and the N5 zone in yellow. Reflection spectrum has been numerically calculated and compared to the reflection measured by spectrophotometer for the N3 and N5 zones, both spectra being normalized and presented in Fig. 7. The reflection spectra show the same optical signature with resonance peaks at substantially the same wavelengths knowing the fabrication allows some inaccuracies regarding the geometric parameters desired. Importantly, these results allow the validation of the simulation model presented in the first part, providing good accuracy of the specific geometrical parameters.

## Conclusion

To conclude, we have simulated, fabricated and optically characterized several designed silver nanorings metasurfaces allowing absorption in the near infrared bands. First, a set of geometric parameters have been predicted by numerical simulation for an enhancement of absorption at optical frequencies. The parametric study of one nanoring but also periodicity studies have been performed. Once this first step was completed, the technical feasibility of its fabrication was tested. A successful design fabrication of different nanorings arrays with several geometric and periodicity parameters has been carried out by a top-down process method, to the point of showing the limits of fabrication with e-beam lithography method. Finally, we validated the FDTD model by comparing the results of optical characterization and numerical simulation of the metasurface. The objective was to perfect the possibilities of absorption of this structure and to facilitate their future design via a new bottom-up technique. Furthermore, the study carried out via FDTD numerical simulation made it possible to optimize the metasurface at visible frequencies and this showed a particular interest for biosensing and therapeutic applications^59^ thanks to the unique properties of split rings resonators metasurface. Actually, these structures have been extensively attracting for biochemical and bio detection research and have been used for instance as dichroic sensors for visible wavelength molecular spectroscopy^15^ or for DNA sensors^60^. As for the optical response at 1500 nm, the applications of interest are related to photodetection with detectors in the SWIR band. Further work will also focus on antisymmetric structures to evaluate the influence of the design but also, from a characterization point of view, to carry out scattering measurements. Indeed, for structures not being symmetrical, the diffusion will depend on the angle of the incident wave and therefore will open new possibilities for optical components which need a large angular acceptance. This research is actually the first step of a future work regarding a new bottom-up method of fabrication of nanorings metasurface made by a self-assembled technique of silver nanocubes into flexible PDMS templates^34^.

## Methods

### FDTD

3D Simulation were conducted using the commercial software FDTD LUMERICAL.

For the results reported in the FDTD simulations sections, simulations conditions were chosen as follows. A plane wave of light is normally incident along the z-axis and the electric field is x-polarized. Symmetric and Perfect Match Layer (PML) boundary conditions are used in x and y directions. PML boundary conditions are chosen in the z direction. The optical constants for silver are extracted from Johnson & Christie’s experimental data^61^. Non-uniform mesh is used.

For the simulation results reported in Fig. 7, simulations conditions were chosen as follows. A plane wave of light is normally incident along the z-axis and the electric field is x-polarized. PML boundary conditions are set on the top and bottom of the computational domain (in the z direction), simulating a highly absorbent material. Periodic conditions are used to repeat periodically the defined pattern along the x and y axis, allowing the simulations of the interactions with neighboring structures. Thus, only a negligible energy would be reflected back in this domain. The optical constants for silver are extracted from Johnson & Christie’s experimental data^61^, from Palik for chromium^43^ and from Ohara for Borosilicate glass^62^. Non-uniform mesh is used. The T detector, allowing the measurement of the transmitted power emitted by the plane wave source is placed 6500 nm below the nanoring composed of chromium and silver.

### Metasurface fabrication

- The glass substrate is cleaned successively in acetone and isopropyl alcohol (IPA) under ultrasonic agitation, dried under clean nitrogen flow and then exposed to oxygen plasma in a barril (Nanoplas France) reactor during 10 min at 150 °C.
- The first layer of highly sensitive positive tone e-beam resist ARP 617.02 (Allresist, Germany) containing a mixture of copolymer on the basis of poly(methyl methacrylate) PMMA and methacrylic acid MMA, safer solvent 1-methoxy-2-propanol, is spin-coated at 6000 rpm during 1 min followed by an annealing at 200 °C for 20 min on a hotplate.
- Then the second layer of ARP 679.02 e-beam resist (solution of 2% PMMA in ethyl lactate) is spin-coated at 6000 rpm during 1 min followed by an annealing at 170 °C for 10 min.
- Then, a conductive resist layer ARPC 5090.02 (Allresist, Germany) is spin-coated at 4000 rpm during 1 min followed by an annealing at 90 °C during 2 min.
- The e-beam lithography is performed by an electron-beam lithography system (PIONEER, Raith, Germany) in order to create the motif of nanorings arrays with the following exposure parameters: acceleration voltage: 20 kV, beam current: 0.018 nA, working distance: 8 mm, nominal dose: 100 µC/cm^2^ and a factor dose: from 1 to 1.2.
- After the exposition by e-beam lithography, the conductive resist is removed by a bath of deionized water for 30 s. Then, the development of the resist is performed during 60 s in a commercial solution AR 600–55 before being stopped in a bath of IPA during 55 s.
- Then, a thin seed layer (3 nm) of chromium is evaporated on the substrate before evaporating a silver layer of 30 nm under vacuum conditions (Auto 306, Edwards, UK).
- Finally, a lift-off process removes the e-beam resists and the excess of silver during some hours in acetone.

The Al_2_O_3_ thin film was grown by ALD in a Fiji 200 reactor (Veeco/Cambridge Nanotech) using trimethylaluminium (Strem Chemicals, 98%) and deionized water. The deposition conditions have been set according to a previous works^63^. The ALD cycle consisted of sequential pulse and purge of TMA and H_2_O in the reaction chamber maintained at 150 °C. The pulse and purge durations were 0.06:10 s for both precursors. In situ spectroscopic ellipsometry was used to adjust the film thickness.

## Supplementary Information

Supplementary Information.

## Data Availability

The data that support the plots within this paper and other finding of this study are available from the corresponding author upon reasonable request.
