# Supplementary material for: Design of infrared optical absorber using silver nanorings array made by a top-down process
Source: Sci Rep. 2023 May 12;13:7770. doi: 10.1038/s41598-023-34579-w (PMC10182000; doi:10.1038/s41598-023-34579-w)
Supplement: Supplementary file 1 — Supplementary Information. [file 41598_2023_34579_MOESM1_ESM.docx]

**Design of infrared optical absorber using silver nanorings array made by a top-down process**

**I. Bouanane ^*ab^, F. Bedu^c^, I. Ozerov^c^, B. Sciacca^c^, L. Santinacci^c^, D. Duché^a^, G. Berginc^b^, L. Escoubas^a^, O. Margeat^c^, J. Le Rouzo^a^**

^a^Aix Marseille Univ, Université de Toulon, CNRS, IM2NP, Marseille, France; ^b^Thales LAS France SAS (France); ^c^Aix Marseille Univ, CNRS, CINAM, AMUTECH, Marseille, France.

# FDTD calculation region

The use of a total-field scattered-field source (TFSF) makes possible the computation region to be separated into two distinct regions (Fig. 1). The first one is the total field region which includes the sum of the incident field wave as well as the scattered field. The second one is the scattered field region which includes only the scattered field. This TFSF source makes possible the study of small nanostructures illuminated by a plane wave. An absorption monitor is located inside the TFSF source. It measures the net absorbed power P flowing through the monitor and, normalizing it to the intensity of the source I, returns the absorption cross section σ_abs._ The scattering cross section σ_sca_, which corresponds also to the scattered power P divided by the power by unit of area of ​​the incident beam I, is calculated thanks to a monitor placed outside the source region.

**Scaterred field region**

**Total field region**

**Simulation region**

**Scattering monitor**

**TFSF source**

**Absorption monitor**

**x**

**z**


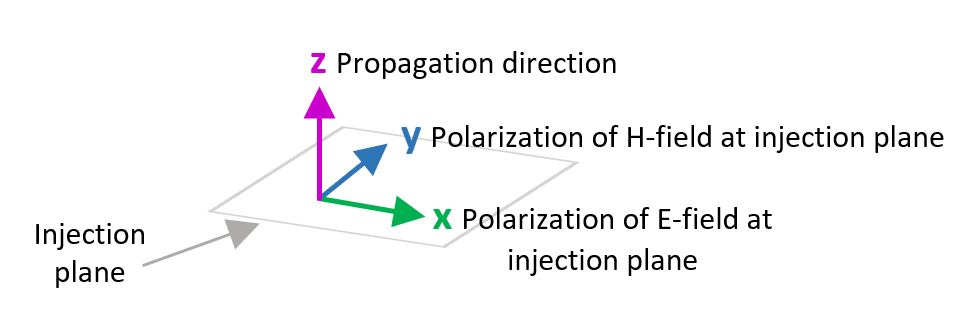


Figure S1: Left: Schematic of the FDTD calculation region. Right: View of the injection plane with the propagation direction and the electromagnetic fields.

Reflection and transmission spectra of the periodic structure composed, here, of a nanoring deposited on a substrate, are calculated with the layout illustrated in Figure 2 thanks to the T and R detectors. Those monitors allow the measurement respectively of the transmitted and reflected power emitted by a plane wave source propagating at normal incidence. Periodic conditions are used to repeat periodically the defined pattern along the x and y axes, allowing the simulations of the interactions with neighboring structures.


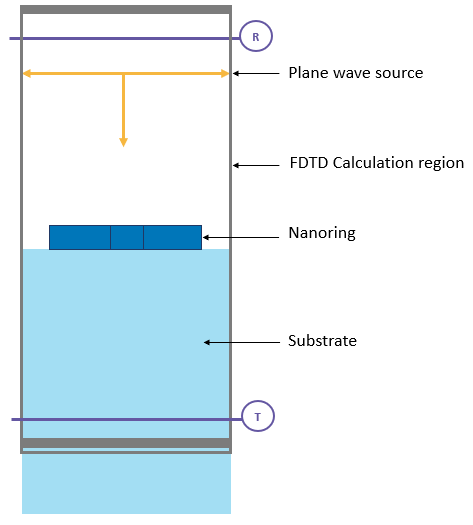


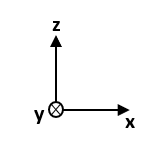


Figure S2: Schematic of the FDTD calculation region composed of a nanoring deposited between a substrate in light blue and an upper media in white which is vacuum. T and R detectors are represented in purple and the plane wave source in yellow.

# FDTD design results

The design results of Figure 2 showing the variation of the absorption and extinction efficiency depending on the geometric parameters of one silver nanoring are summarized in Figure S3. It shows the variation of the resonant frequency and of the absorption in function of the geometric parameters “z” in Fig. S2 a) and b), “(h_2_-h_1_) = z” in Fig. S2 c) and d) and of the absorption and extinction in function of the parameter “g” in Fig. S2 e), f), g) and h).


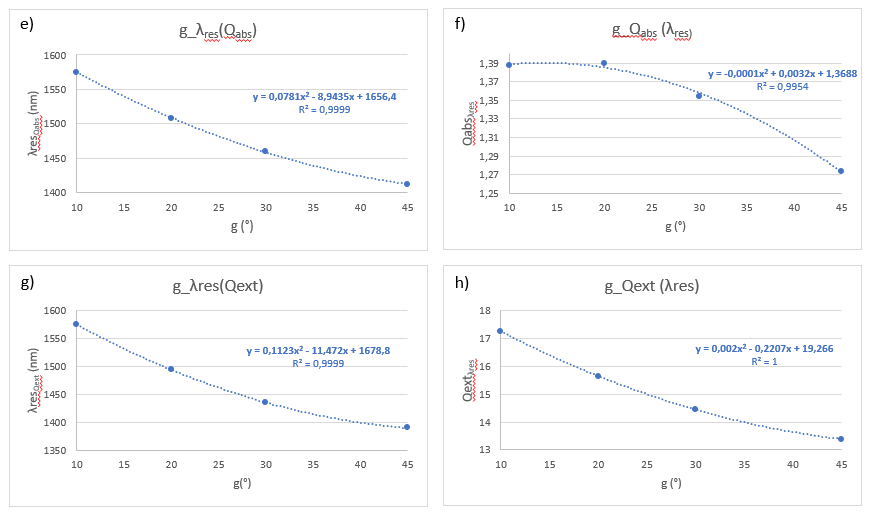

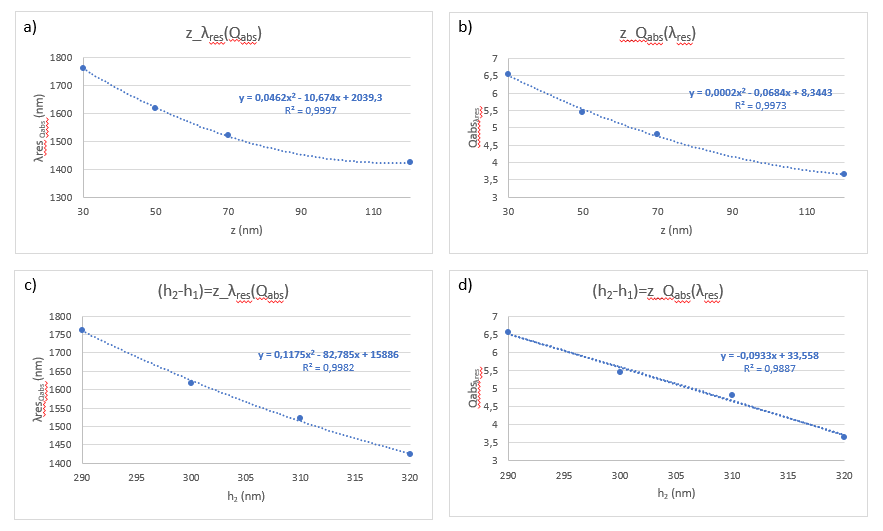


Figure S3: Fitting with polynomial regressing the variation of the resonant frequency and the absorption efficiency, showing in Figure 2, in function of the geometric parameters “z” a) and b), “z=h2-h1” c) and d), and the gap “g” e) and f).

# Polarization study


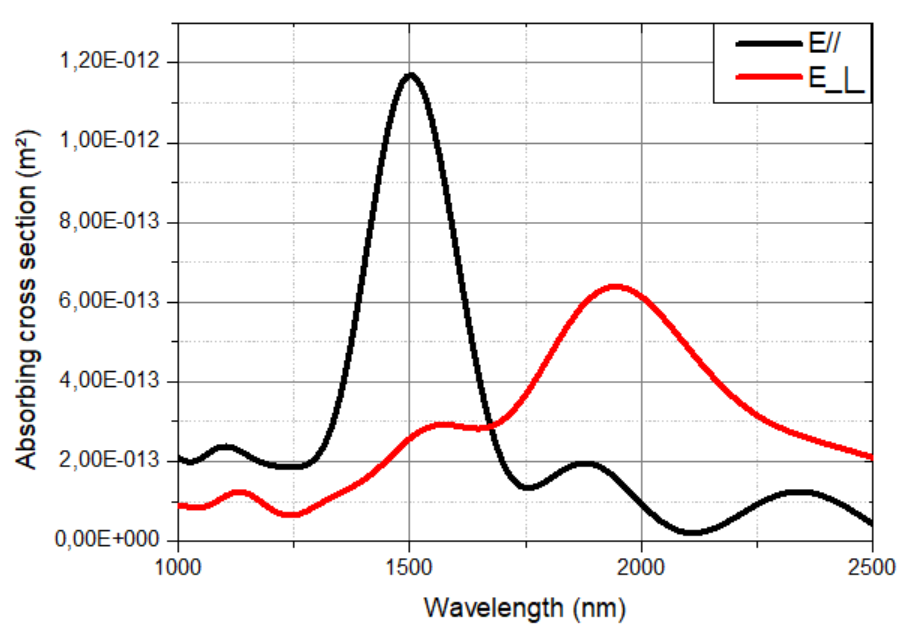

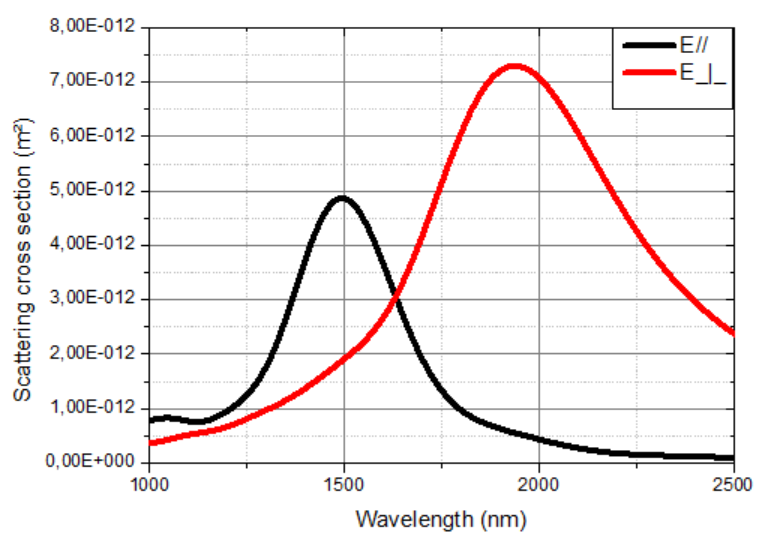


d)

b)

c)

a)


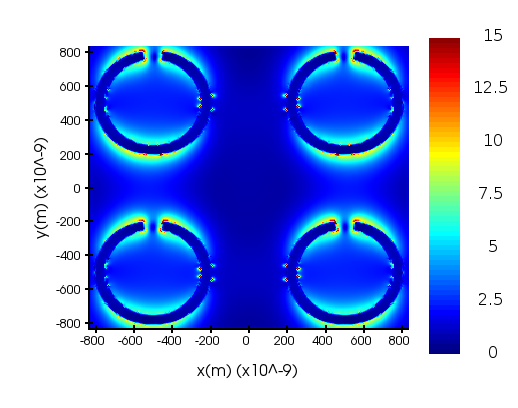


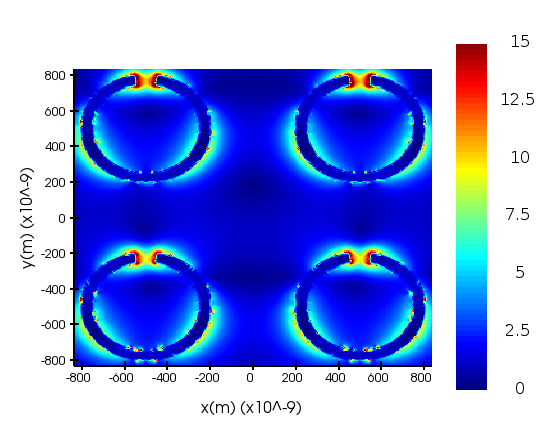

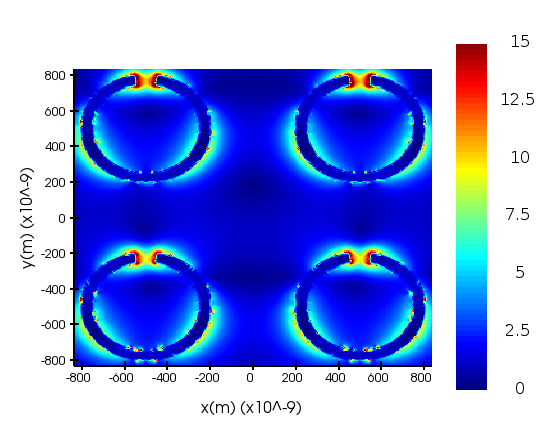


Figure S4: Electromagnetic simulation of polarization dependence for four nanorings separated by 390nm : a) Absorbing cross section b) Scattering cross section. Mapping of the electric field intensity: c) at 1500 nm for the parallel polarization d) at 1950 nm for the perpendicular polarization.

# Silver nanoring fabrication

The main steps of the fabrication method are here after. First, a transparent substrate of borosilicate glass with a refractive index of 1.5 at a wavelength of 587 nm has been chosen and is cleaned both chemically and by plasma. Before the e-beam lithography and the creation of the pattern, two e-beam resist of methyl methacrylate (MMA) and poly(methyl methacrylate) (PMMA) layers as well as a conductive resist were deposited on the substrate by spin-coating. After the development of those resists, the mask of the nanorings array is obtained by evaporation of a thin layer of chromium and a thicker layer of silver. The final step is the lift-off process which is a key step of this fabrication process for pattern transfer. Indeed, this stage is well known to be unstable and unreliable, that is why a double layer of resists has been deposited by spin coating before e-beam lithography, as shown in Figure 5 of the Supplementary Note. This favors the lift-off stage and the deposition of a thicker layer of metal.


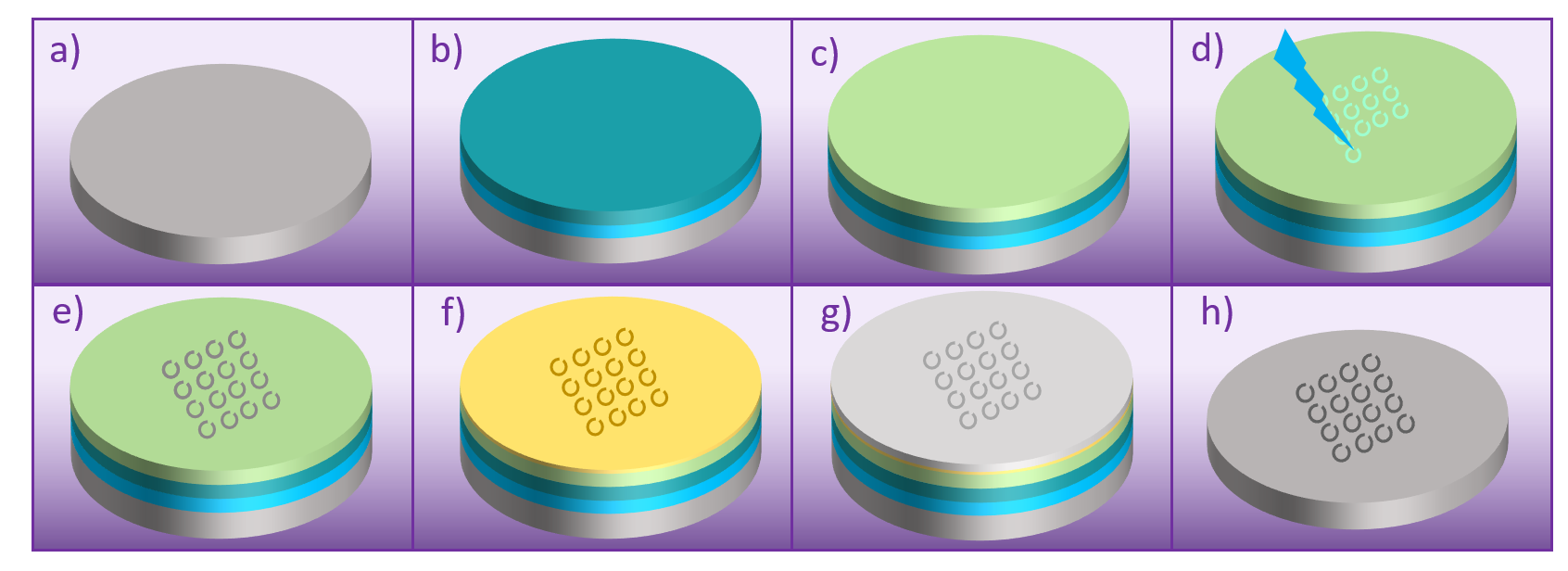


Figure S5: Schematic of the fabrication method of the silver split-rings metasurface

a) A borosilicate glass substrate is cleaned chemically and by plasma; b) Spin-coating of the e-beam resists (MMA + PMMA); c) Spin coating of the conductive protective resist; d) E-beam lithography; e) Development of the resists; f) Evaporation of a thin chromium layer; g) Evaporation of a silver layer; h) Lift-off of the resist


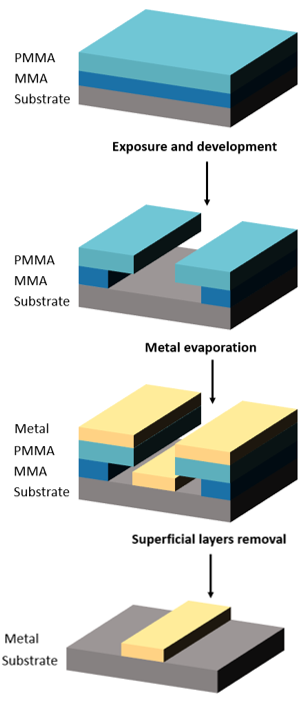


Figure S6: Schematic of the double resists process


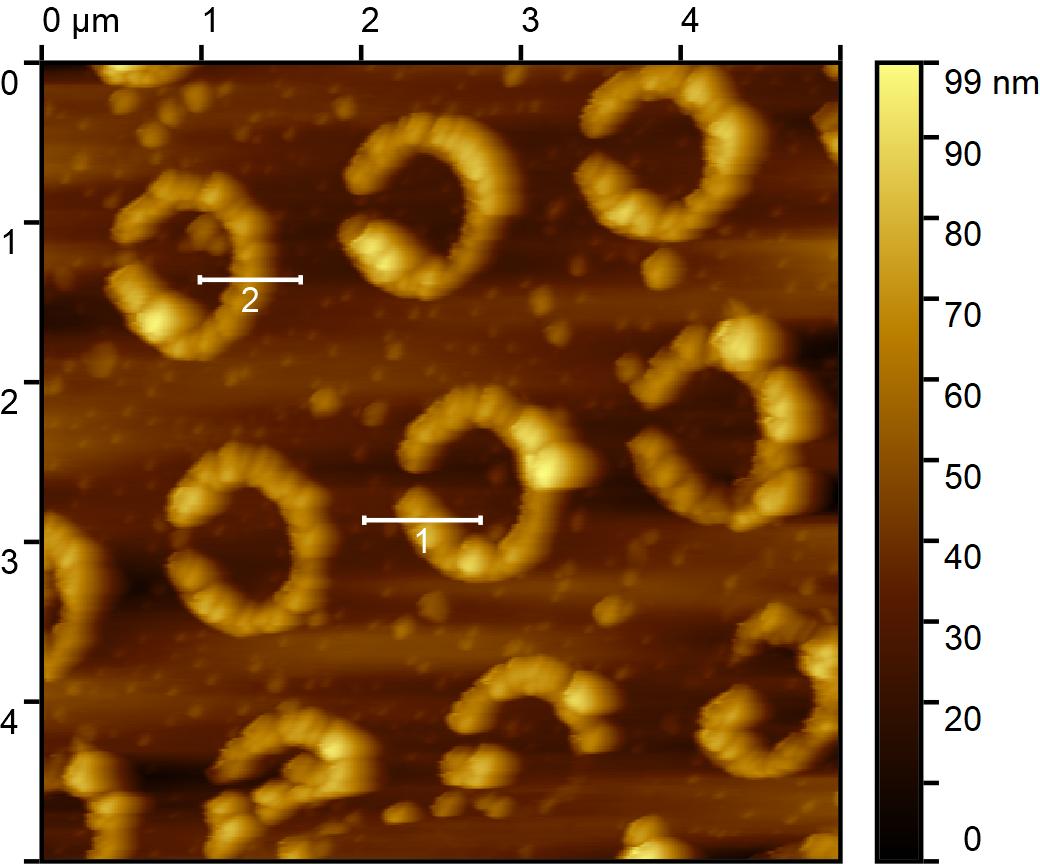


a)

b)


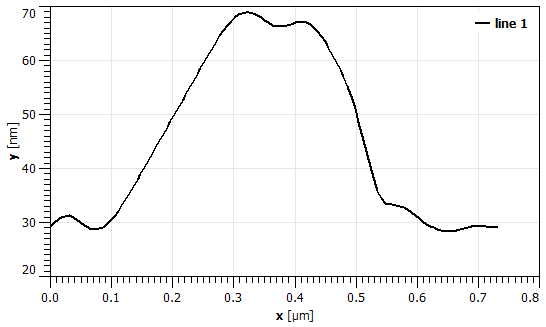


Figure S7: a) AFM images of the N2 fabricated design zone and b) the measure of the thickness difference of the line 1 of the AFM image.





Figure S8: Fabricated periodic structure on a large surface : SEM image of the array N5


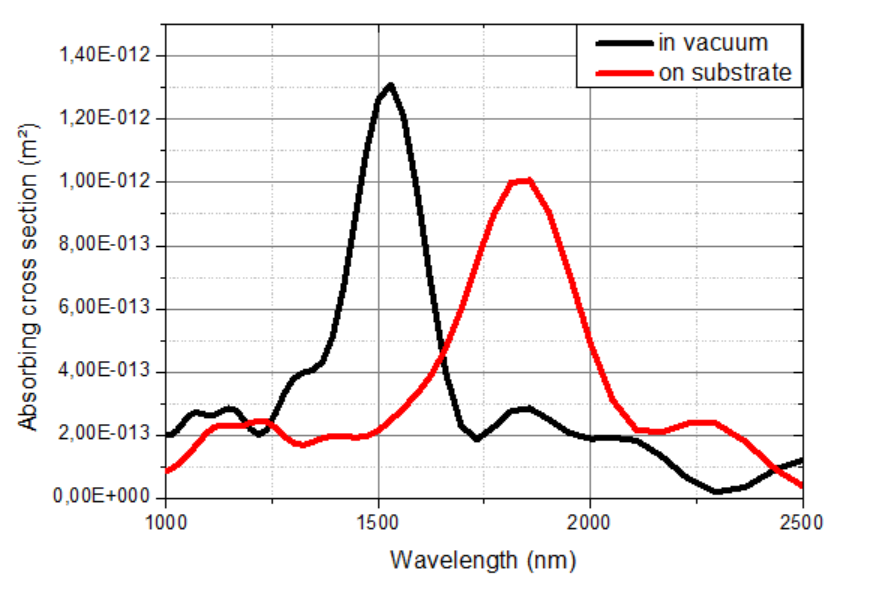

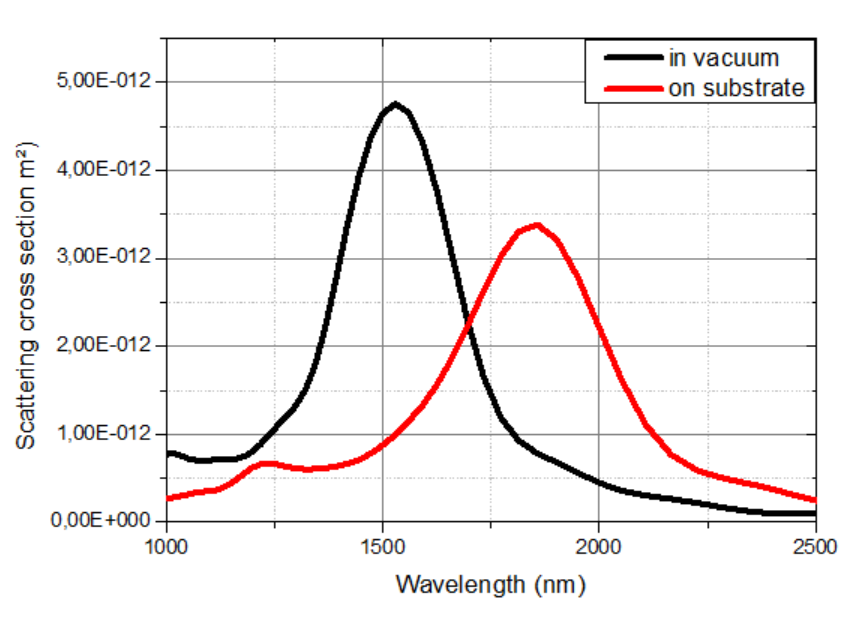


a)

b)

Figure S9: Wavelength-dependent absorbing (a) and scattering (b) cross section of four nanorings simulated in vacuum in black and on a glass substrate with a thin layer of 3nm of Chromium in red.

a)

b)


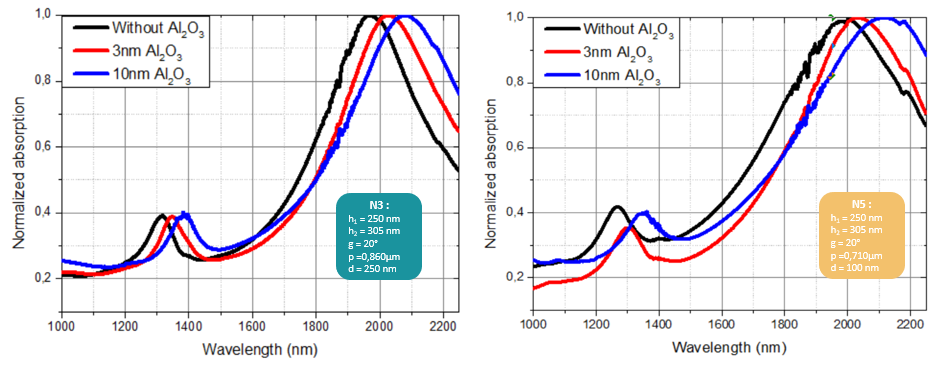


Figure S10: Wavelength-dependent normalized absorption measured with no layer of alumina in black, a layer of 3 nm in red and 10 nm in blue, for N3 (a) and N5 (b).
